# The Phenotypic Analysis of *Lactobacillus plantarum shsp* Mutants Reveals a Potential Role for *hsp1* in Cryotolerance

**DOI:** 10.3389/fmicb.2019.00838

**Published:** 2019-04-24

**Authors:** Mattia Pia Arena, Vittorio Capozzi, Angela Longo, Pasquale Russo, Stephanie Weidmann, Aurélie Rieu, Jean Guzzo, Giuseppe Spano, Daniela Fiocco

**Affiliations:** ^1^Department of Agriculture, Food and Environment Sciences, University of Foggia, Foggia, Italy; ^2^Univ. Bourgogne Franche-comté, AgroSup Dijon, PAM UMR A 02.102, Dijon, France; ^3^Department of Clinical and Experimental Medicine, University of Foggia, Foggia, Italy

**Keywords:** small heat shock proteins (sHSP), chaperone, *Lactobacillus plantarum*, probiotic, stress, membrane fluidity

## Abstract

Small heat shock proteins (sHSPs) are ubiquitous, low molecular weight (MW) proteins that share a conserved alpha-crystallin domain. sHSPs oligomers exhibit chaperon-like activities by interacting with unfolded substrates, thereby preventing their aggregation and precipitation. Unlike most lactobacilli, which have single *shsp* genes, three different sHSP-encoding genes, i.e., *hsp1*, *hsp2*, and *hsp3*, were previously identified in the probiotic *Lactobacillus plantarum* WCFS1. Early studies, including the characterization of the knock out (KO) mutant for *hsp2*, indicated a different organization and transcriptional regulation of these genes and suggested that the three *L. plantarum* sHSPs might accomplish different tasks in stress response. To unravel the role of sHSPs, KO mutants of *hsp1* and *hsp3* were generated using a Cre-*lox* based system. Mutation of either genes resulted in impaired growth capacity under normal conditions, heat-stress and stresses typically found during host interactions and food technological process. However, survival to heat shock and the level of thermal stabilization of cytoplasmic proteins were similar between mutants and parental strain. Transcriptional analysis revealed that in the mutant genetic backgrounds there is an upregulated basal expression of the un-mutated mate *hsps* and other stress-related genes, which may compensate for the loss of HSP function, hence possibly accounting for the lack of a remarkable susceptibility to heat challenge. HSP3 seemed relevant for the induction of thermotolerance, while HSP1 was required for improved cryotolerance. Cell surface properties and plasma membrane fluidity were investigated to ascertain the possible membrane association of sHSP. Intriguingly, the loss of *hsp1* was associated to a lower level of maximal membrane fluidity upon heat stress. A role for HSP1 in controlling and improving membrane fluidity is suggested which may pertains its cryoprotective function.

## Introduction

Small heat shock proteins (sHSPs) are ubiquitous, low molecular weight (MW; 12–43 kDa) ATP-independent chaperones that share a signature alpha-crystallin domain of about 100 amino acids (Haslbeck et al., 2019). sHSPs are intimately linked to protein homeostasis and play a protective function under stress conditions, especially for survival to heat shock. In response to stress signals, sHSPs are upregulated and, as demonstrated by *in vitro* studies, assemble into dynamic oligomeric structures that bind denatured and/or partially unfolded polypeptides, thereby preventing their irreversible aggregation (Nakamoto and Vigh, 2007). Because sHSP are “holdase” chaperones, their client proteins are subsequently released to other downstream ATP-dependent chaperones, which can actively re-fold them to their native state (Eyles and Gierasch, 2010; Carra et al., 2017).

Though sHSPs are mainly cytosolic, some of them have been shown to be membrane-associated. Such localization has been related to a peculiar lipochaperone function, i.e., their ability to bind membrane lipids, thus modulating membrane fluidity and organization. To date, this membrane-stabilizing effect has been demonstrated for only a few sHSPs, including those from microbial species which inhabit very hostile and/or variable environments, and thus seem to have evolved specific strategies to cope with harsh and changing conditions (Török et al., 2001; Maitre et al., 2012).

Lactobacilli are natural members of the gut microbiota in mammals, including humans, and many commensal species have gained attention for their positive impact on host health (van Baarlen et al., 2013). *Lactobacillus plantarum*, a member of the lactic acid bacteria (LAB) group, is among the most common lactobacilli occurring on the human oral, vaginal and intestinal mucosa (De Vries et al., 2006; Arena et al., 2017). *L. plantarum* boasts a documented history of safe use in food, as it has been traditionally employed for the artisanal and industrial production of several fermented foods, where it is either naturally present or added as a starter to drive the fermentation process (Mogensen et al., 2002). Moreover, diverse *L. plantarum* strains have been claimed health-promoting properties and are useful for the preparation of currently commercialized probiotic formulations (Bove et al., 2012b; Seddik et al., 2017). Being remarkably widespread in the environment, this bacterium is a natural inhabitant of the human gastrointestinal tract and occurs in several foods fermentation-associated niches, where diverse stresses, such as heat, cold, and acidity, are frequent. In contrast to most probiotic lactobacilli, which usually have one or two *shsp* genes (Capozzi et al., 2011a), three sHSP-encoding genes, i.e., *hsp1*, *hsp2*, and *hsp3* (also referred to as *hsp18.5*, *hsp18.55*, and *hps19.3*, respectively) have been identified in the probiotic reference strain *L. plantarum* WCFS1 (Kleerebezem et al., 2003; Spano et al., 2004, 2005). Such redundancy might endow *L. plantarum* with a distinctive capacity to cope with a broad range of stresses, thus accounting for its extraordinary adaptability.

Previous studies, including *in silico* and functional analyses of the *hsp* promoters, evaluation of the expression pattern, and homologous over-production strategies (Spano et al., 2005; Fiocco et al., 2007, 2010; Bove et al., 2011; Russo et al., 2012), hinted to a different genetic regulation and suggested that the three sHSPs might accomplish different tasks in the stress response of *L. plantarum*. Furthermore, deletion of *hsp2* did not significantly impair resistance to heat and other stresses, while it was shown to affect cell morphology and plasma membrane fluidity, even under normal growth conditions (Capozzi et al., 2011b). Therefore, it was suggested that such chaperone might fulfill a housekeeping role with a general protective function for proteome homeostasis.

In the present study, we have generated and phenotypically characterized *L. plantarum* WCFS1 knock out (KO) mutants for *hsp1* and *hsp3*, in an attempt to understand the contribute of these sHSP to the stress response in this model probiotic.

## Materials and Methods

### Bacterial Strains and Growth Conditions

The bacterial strains used in this work are listed in Supplementary Table S1. *Escherichia coli* strain DH10B was used for DNA cloning experiments and was grown in Luria-Bertani (LB) broth at 37°C, with shaking. *L. plantarum* WCFS1 and its derivative mutants were grown in De Man Rogosa Sharpe (MRS, Liofilchem, Italy) broth (pH 6.2), at 30°C, without shaking. Agar (15 g L^-1^) was added to obtain solid media. For the selection of recombinant strains, antibiotics were added to the growth medium at the following concentrations: 100 μg mL^-1^ ampicillin and 200 μg mL^-1^ erythromycin, for *E. coli*; 10 μg mL^-1^ erythromycin and 10 μg mL^-1^ chloramphenicol for *L. plantarum*.

The growth rates of the *hsp1* and *hsp3* mutant and wild-type *L. plantarum* strains were determined by diluting overnight cultures to an optical density, at 600 nm (OD_600_), of 0.0125 in fresh MRS medium. The turbidity of the cultures was monitored by measuring OD_600_ at 30 min intervals, in an EON Microplate Spectrophotometer (Biotek, VT, United States), over a complete growth cycle (18–20 h), at 30°C (control) or at 42°C (heat stress condition), in unsupplemented MRS (control) or in MRS containing diamide (4, 2, 0.5 mM, i.e., oxidative stress), ethanol [4, 6%, (v/v)], bile salts [0.05, 0.1, 0.2% (w/v)], NaCl [4, 6, 8% (w/v), i.e., hyperosmotic stress], or in HCl-acidified MRS (pH 4.0, 4.5). Maximum growth rate values (i.e., μ_max_) were calculated as the maximal slope of the growth curves reporting lnOD_600_ as a function of time, using the following formula: μ_max_ = (lnOD_f_-lnOD_0_)/(t_f_-t_0_); OD_f_, final optical density; OD_0_, initial optical density; t_f_, final time; t_0_, initial time (Ferrando et al., 2015). Duration of lag phases was estimated by extrapolating the tangent at the exponential part of the growth curve (Hall et al., 2014).

### DNA Manipulation

DNA isolation, endonuclease treatment and ligation, were performed as previously described (Fiocco et al., 2009). *Taq* polymerases, restriction endonucleases, alkaline phosphatase, and T4 DNA ligase were purchased from Roche (Milan, Italy), Invitrogen (Milan, Italy), New England Biolabs (Hertfordshire, United Kingdom), Fermentas (Burlington, ON, Canada), and Promega (Milan, Italy) and were used as recommended by the suppliers.

Plasmids were isolated using QIAprep spin miniprep kits (Qiagen, Hilden, Germany). PCR products and DNA restriction fragments were purified with the QIAquick PCR purification and gel extraction kits (Qiagen). *L. plantarum* genomic DNA was isolated using a microbial DNA extraction kit (Cabru, Milan, Italy) according to the manufacturer’s procedure. *L. plantarum* genomic DNA (approximately 20 ng) was PCR amplified using Expand Long Template kit (Roche) following the manufacturer’s instructions.

### Generation of *hsp1* and *hsp3* Mutants, i.e., KO1 and KO3 Derivative Strains

The *L. plantarum* mutants were obatined using a previously developed vector system (Lambert et al., 2007). The chromosomal regions upstream (850-bp fragment; primer pair FB1 hsp1–RB1 hsp1, Supplementary Table S1) and downstream (960-bp fragment; primer pair FB2 hsp1–RB2 hsp1) of *hsp1* were PCR-amplified using a proofreading DNA polymerase and *L. plantarum* WCFS1 chromosomal DNA as a template. The amplicons were digested with Ecl136II and SwaI, respectively, and cloned into SwaI and Ecl136II restriction sites of the suicide vector pNZ5319 (Lambert et al., 2007) to give the recombinant mutagenesis vector pNZ5319-KOhsp1 (Supplementary Table S1). Likewise, the regions upstream (870-bp; primer pair FB1 hsp3–RB1 hsp3) and downstream (900-bp; primer pair FB2 hsp3–RB2 hsp3) of *hsp3* were PCR-amplified, digested with XhoI-SwaI and SwaI, respectively, and cloned into XhoI-SwaI and Ecl136II restriction sites of pNZ5319, to give the recombinant mutagenesis vector pNZ5319-KOhsp3. pNZ5319-KOhsp1 and pNZ5319-KOhsp3 were electroporated into *L. plantarum* WCFS1 (MicroPulser electroporation apparatus, BioRad, Hercules, CA, United States). Chloramphenicol-resistant transformants were selected and replica-plated to check for erythromycin sensitivity, reflecting double cross-over events and loss of the suicide vector. Candidate double-crossover mutants were analyzed by colony PCR. Correct integration of the *lox66-P32-cat-lox71* cassette into the genome was verified by PCR on isolated chromosomal DNA using primers annealing to external genomic regions from those cloned (i.e., Hsp1/Hsp3-B1ext-FOR, Hsp1/Hsp3-B2ext-Rev) combined with *cat* cassette specific primers (i.e., CAT For, CAT Rev) (Supplementary Table S1). DNA sequencing of target genomic regions confirmed *hsp1* and *hsp3* disruption. The *hsp1* and *hsp3* mutants were named KO1 and KO3, respectively (Supplementary Table S1).

### Protein Aggregation Assay

Crude cell extracts were obtained by glass beads homogenization (Fiocco et al., 2007) from *L. plantarum* cultures (OD_600_≈ 1) grown at 30°C with or without shifting to 40°C for 1 h prior extraction. Briefly, cells were washed in saline water (9 g L^-1^ NaCl) and subjected to four rounds of mechanical disruption in ice–cold sodium phosphate buffer (50 mM, pH 7.0). The extracts were ultracentrifuged at 68,600 × *g* for 1 h at 10°C and the supernatant fractions, containing cytoplasmic proteins, were collected. The protein concentration was adjusted to 2 mg mL^-1^. Protein samples were incubated at 55°C for 30 min and subsequently centrifuged at 16,000 × *g* for 10 min. The amounts of proteins in the pellet and supernatant fractions, corresponding to aggregated and soluble proteins, respectively, were determined by the Bradford dye^-^binding method (Bio-Rad protein assay, Bio-Rad, Hercules, CA, United States).

### Analysis of Physicochemical Cell Surface Properties

The microbial adhesion to solvents (MATS) method (Bellon-Fontaine et al., 1996) was used to determine the hydrophobic and acid-base characters of the cell surface, as previously reported (Bove et al., 2012a). Briefly, overnight cultures of *L. plantarum* strains were harvested by centrifugation at 5,000 × *g* for 10 min, washed twice, and resuspended to an OD_600_ of 1 (A_0_) in PBS. In glass tubes, 2.4 mL of bacterial suspension were mixed by 2 min of vortexing with 0.4 mL of solvent. After 15 min incubation at room temperature to allow phase separation, the aqueous phase was collected, and its OD_600_ was measured (A_1_). The percentage of bacterial adhesion to solvent was calculated as [1 - (A_1_/A_0_)] × 100. The solvents used were: hexadecane (HE), chloroform (CH) and ethyl acetate (EA).

### Biofilm Assay

Biofilm assays were performed as described in Bove et al. (2012a), with minor modifications. *L. plantarum* strains were cultured in 24-well cell culture plate (Costar, Corning, NY) for 2–7 days. Wells were washed twice and the adherent biofilms were stained with 2 mL 0.1% (w/v) crystal violet for 5 min. After rinsing three times, the biofilms were solubilized in 2 mL of 30% (v/v) acetic acid and optical density at 570 nm (OD_570_) was measured.

### Tolerance to Heat Shock

Tolerance to heat shock was studied following or not a thermal adaptation phase. Overnight cultures were diluted to OD_600_ = 0.01 into MRS and allowed to grow at 30°C until OD_600_ = 0.8. After 30 min of further incubation at 30 or 40°C (adaptation), cultures were shifted to 55°C in a water bath for 25 min. Immediately after the thermal shock, bacterial samples were cooled. The number of viable cells was determined before and after heat shock as colony forming units (CFU)/mL by plating serial dilutions on MRS agar plates. The death index, defined as log (N_0_/Ns) (N_0_, initial cell count; Ns, cell count after stress) was calculated. The experiment was repeated at least three times.

### Freeze-Thaw Challenge

Cultures from logarithmic (OD_600_ = 0.7–0.8) and stationary growth phase were frozen at -30°C in their medium without adding any cryoprotective agent. Every 48 h, the samples were thawed at room temperature, to allow dilution and plating, and then frozen again. A total of 7 freeze-thaw cycles was performed. For each sample, plated CFU were determined before the first freezing and, after thawing, at each freeze-thaw cycle. The death index was calculated as above. The experiment was repeated on three independent cultures for each strain.

### Fluorescence Anisotropy Measurements

Membrane fluidity was examined according to Capozzi et al. (2011b), with some modifications. Exponential phase cells (20 ml culture at an OD_600_ of 0.6–0.7) were harvested by centrifugation (6,300 × *g*, 10 min) and washed three times in 20 ml of 50 mM MES {[2-(N-morpholino) ethanesulfonic acid]-KOH buffer (pH 6.2)} containing 10 mM glucose. The cell pellet was resuspended in the same buffer. The OD_600_ of the cell suspension was adjusted to 0.7 in all measurements. The cell suspension was immediately used for fluorescence anisotropy measurements.

Membrane fluidity was determined continuously by measuring fluorescence anisotropy in intact whole cells by using hydrophobic 1,6-diphenyl-1,3,5-hexatriene (DPH) (Molecular Probes) as a probe and a stirred and thermoregulated cell holder of the spectrofluorometer (FLUOROLOG-3, Jobin Yvon, Inc., United States) in a T format. Excitation and emission wavelengths were 352 and 402 nm, respectively. Fluorescence anisotropy (which is inversely proportional to membrane fluidity) was measured with determinations made every 8 s. The temperature of cell suspensions was automatically detected by the Peltier system. 8 μL of a 1.5 mM DPH solution was added to 2.5 mL of cell suspension to achieve the final probe concentration of 4.0 μM. To ensure probe stabilization for optimal anisotropy determinations, cells were incubated with the DPH probe in 50 mM MES-KOH buffer, 10 mM glucose, pH 6.2, at 28°C for 10 min before shock and labeled cells were then directly stressed during the measurement. Instantaneous heat shock was performed by adding 500 μL of a preheated (44°C) 50 mM MES-KOH buffer, 10 mM glucose, pH 6.2, to a concentrated cell suspension to achieve the OD_600_ of 0.6 in all measurements. Each experiment was performed three times from independent cultures.

### Gene Expression Analysis

Total RNA was extracted from *L. plantarum* in order to analyze gene expression at the transcriptional level. In detail, RNA was isolated from wild type (WT) and *hsp* mutants log cultures (OD_600_ = 0.8) under normal growth conditions (i.e., in unsupplemented MRS at 30°C) and after thermal upshift (30 min and 1 h, at 42°C). The UltraClean microbial RNA isolation kit (Cabru, Milan, Italy) was used to obtain total RNA. RNA quality and concentration were estimated by gel electrophoresis and spectrophotometrically (BioTek Instruments, Winooski, VT, United States). Total RNA (approximately 0.5 μg) was reverse transcribed using the QuantiTect Reverse Transcription kit (Qiagen), which includes a DNase I treatment. The transcriptional level of target genes was analyzed by qRT-PCR in a real-time PCR instrument (ABI 7300; Applied Biosystems, Foster City, CA, United States), using SYBR green I, in three biological replicates. The PCR reaction, including 5 μL of 20-fold diluted cDNA, 10 μL of QuantiFast SYBR Green PCR Master Mix (Qiagen) and 250 nM of each forward and reverse primer, was cycled for 5 min at 95°C, followed by 35–40 cycles of 10 s at 95°C and 30 s at 60°C (i.e., annealing and extension phase). The absence of chromosomal DNA contamination was verified by real-time PCR on corresponding DNase I-treated RNA. After each run, melting-curve analyses confirmed specific amplification. Fluorescence data were analyzed by applying the ΔΔCt method (Livak and Schmittgen, 2001). Target stress response genes included those coding for small heat shock proteins, i.e., *hsp1* (lp_0129), *hsp2* (lp_2668), *hsp3* (lp_3352), for members of the ATP-dependent Clp protease system, including the ATP-binding subunits ClpB (*clpB*, lp_1903), ClpC (*clpC*, lp_1019), ClpE (*clpE*, lp_1269) and the proteolytic subunit ClpP (*clpP*, lp_0786), for the ATP-dependent zinc metallopeptidase FtsH, i.e., *ftsH* (lp_0547), for the GroEL chaperonin (*groEL*, lp_0728) and the DnaK chaperone, i.e., *dnaK* (lp_2027). The transcript levels of the housekeeping genes coding for lactate dehydrogenase D (*ldhD*) and TU elongation factor (*tuf*) were used as internal controls. Such genes were selected following a preliminary evaluation of *ldhD* and *tuf* Ct fluctuations, under the tested conditions, and according to previous studies supporting their expression stability and suitability for similar applications (Fiocco et al., 2008; Arena et al., 2011; Bove et al., 2013). The complete list of the oligonucleotides used in this study is reported in Supplementary Table S1.

### Statistical Analysis

The statistical significance of the differences observed was determined using one-way ANOVA and two-tailed Student *t*-tests, with *p* < 0.05 as the minimal level of significance. Only for the MATS assay, the confidence interval was extended to 90%, i.e., *p* < 0.1 as the minimal level of significance.

## Results

### Knock Out of *hsp1* and *hsp3* Genes Affects Growth Capacity Under Both Optimal and Stress Conditions

**FIGURE 1:**
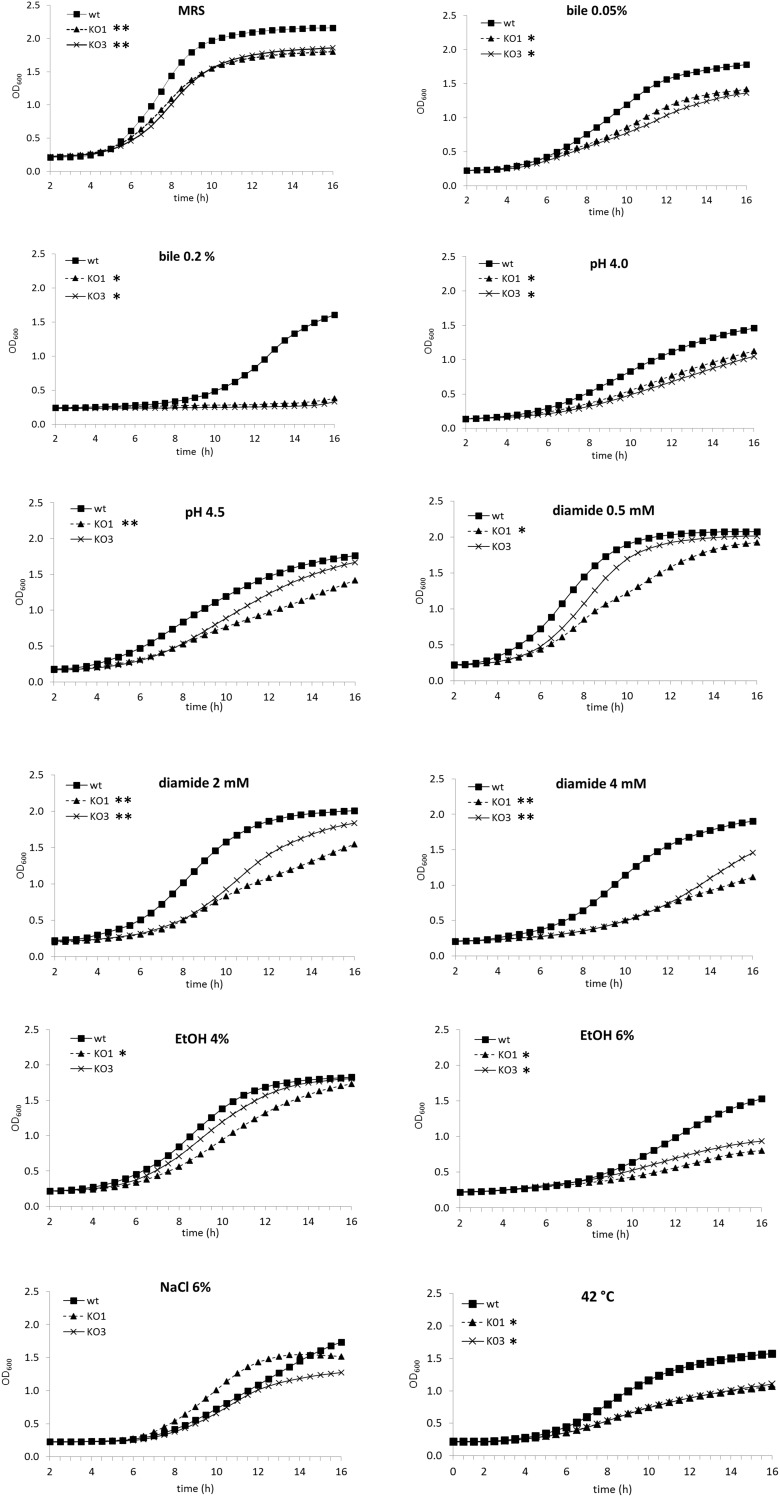
Growth curves of *Lactobacillus plantarum* wild type (WT) and derivative mutant strains. The optical density at 600 nm (OD_600_) was monitored over a 16 h period for cultures of WT, solid square, *hsp1* mutant (KO1), solid triangle, and *hsp3* mutant (KO3), crosses, under optimal growth conditions at 30°C in unsupplemented MRS broth (pH 6.2), in acidified medium, or with the addition of different chemical stressors, or under heat stress (incubation at 42°C). Values are mean from at least two independent experiments run in triplicates (SD was omitted for improved clarity). The asterisks indicate the mutant growth curve with significantly different OD_600_ values (*P* ≤ 0.05) from that of WT for over 50% (^∗∗^) or 30% (^∗^) of the reported time points, as assessed by Student *t*-test.

The growth abilities of WT, *hsp1* (KO1) and *hsp3* (KO3) mutant strains was monitored by turbidity and investigated under optimal growth conditions and under stresses which are typically associated to biotechnological applications and probiotic lifestyle, i.e., presence of bile salts, high acidity, oxidative environment, hyperosmotic medium, ethanol stress and heat (Figure 1 and Supplementary Table S2). Under optimal growth conditions (unsupplemented MRS), the growth of both mutants was inhibited compared to WT. In presence of bile 0.05%, the WT growth was impaired, but growth inhibition was more evident for the mutants; this negative effect was particularly strong for the mutants at a higher bile concentration (i.e., 0.2% w/v), which was associated to prolonged lag times and strongly reduced growth rates (Supplementary Table S2). In acidified medium (pH 4.5 and 4.0), the growth of all clones was negatively affected, most consistently for the mutant strains and, particularly, for KO1. Likewise, under oxidative conditions (i.e., presence of diamide), growth of both mutants, especially KO1 mutant, was impaired. In presence of 4% ethanol (v/v), KO1 growth was much affected compared to WT, exhibiting longer lag time and slower growth. Both mutants exhibited a similarly, impaired growth at higher ethanol concentration (6%, v/v). Under hyperosmotic conditions (NaCl 8%, w/v), the growth was significantly reduced for all clones (data not shown); at NaCl 6% (w/v), WT would grow slowly and similarly, to KO3, while KO1 growth was the least weakened. When cultivated at a suboptimal temperature of 42°C, the growth kinetics of KO1 and KO3 were similar to each other and strongly diminished relative to WT (i.e., with similar lag times, but much lower maximal growth rates).

### Effect of *hsp1* or *hsp3* on the Heat Stress Tolerance and Thermal Stabilization of Cell Proteins

**FIGURE 2:**
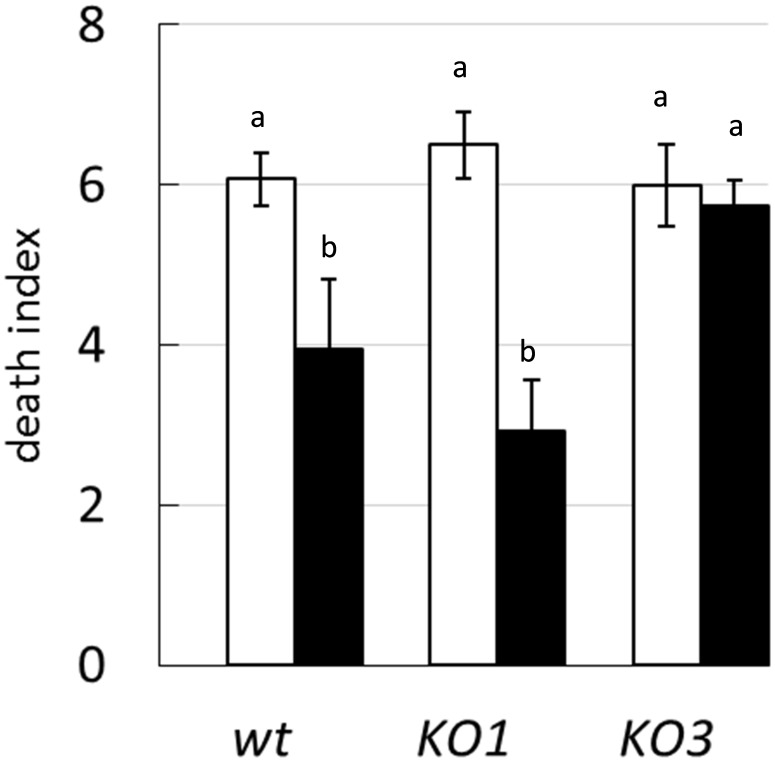
Survival to heat shock. The death index of *L. plantarum* WCFS1 WT and its derivative mutants for *hsp1* (KO1) and *hsp3* (KO3) was determined by comparing the viable counts before and after 25 min incubation at 55°C, with (solid bars) or without (open bars) a thermal pre-adaptation step (i.e., 30 min incubation at 40°C). Data are mean and SD of three independent experiments. Different letters indicate statistically significant differences (*P* ≤ 0.05), as determined by ANOVA test.

To evaluate the relevance of *hsp1* and *hsp3* in heat shock resistance and in the induction of thermotolerance, the survival of WT and mutant strains was analyzed upon exposure to a lethal temperature of 55°C, following or not a thermal pre-adaptation at a sublethal temperature of 40°C. Shifting directly WT cultures to 55°C resulted in a 6 log-decrease of viability. A similar death rate was observed also for both the mutants (Figure 2). In WT cultures pre-exposed to 40°C, the loss of viability (after upshift to lethal temperature) was significantly reduced (4 log decrease), compared to non-pre-exposed cultures. Likewise, a greater survival was observed for pre-adapted *hsp1* mutant cultures. Whereas the survival of the *hsp3* mutant was not influenced.

**FIGURE 3:**
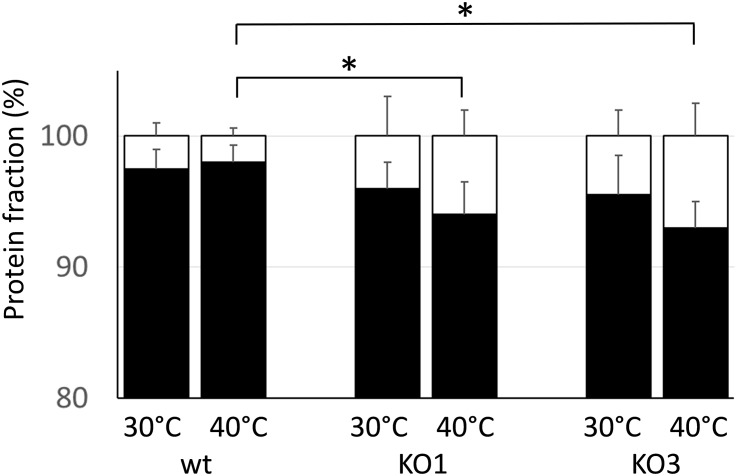
Thermostabilization of cell proteins. Protein extracts from WT and mutant clones (KO1 and KO3) were obtained from cultures either grown at 30°C or grown at 30°C and upshifted to 40°C for 30 min prior protein extraction. After heating the protein samples at 55°C for 30 min, the amount of aggregated (white) and soluble (black) proteins was determined and expressed as a percentage of the total proteins. Data are mean and SD of two independent experiments with two replicates. ^∗^Statistically significant difference relative to WT (*P* ≤ 0.05; Student *t*-test).

Because one of the main functions of sHSP is to prevent the aggregation of cytoplasmic proteins under heat challenge, the level of aggregated proteins was examined in cell extracts upon exposure to a denaturing temperature (55°C) (Figure 3). The percentage of aggregated proteins was low (ranging 3–5%) and it did not significantly differed between samples obtained from WT and mutants. When cultures were pre-cultivated at a sublethal high temperature, the aggregated fraction slightly increased in both mutants, compared to WT.

These data indicate that the lack of either HSP1 or HSP3 does not worsen survivability to an intense heat shock, nor has any dramatic effect on the ability to prevent aggregation of cytoplasmic proteins. Moreover, it appears that *L. plantarum* can acquire increased heat resistance upon thermal pre-adaptation, though the KO3 mutant is unable to develop such property.

### *hsp1* Is Required for Improved Cryotolerance

**FIGURE 4:**
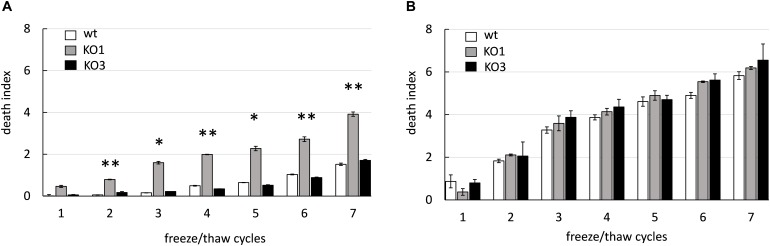
Cryotolerance of *L. plantarum* WCFS1 WT and its derivative mutants. The death index of (WT, open bars), *hsp1* mutant (KO1, gray bars), and *hsp3* mutant (KO3, solid bars) from log **(A)** and stationary **(B)** phase cultures was determined by comparing the viable counts before and after freezing and it is reported as a function of the number of freeze-thaw cycles. Data are mean and SD from 2 independent experiments runs in triplicates. Statistical significance relative to WT values, ^∗∗^*P* ≤ 0.01; ^∗^*P* ≤ 0.05 (Student *t*-test).

The potential role of sHSP in cryotolerance was investigated by assessing viability of WT and KO1 and KO3 mutants upon freeze-thaw cycles (Figure 4). As expected, the viability of all cultures tended to decrease as a function of the number of freeze-thaw cycles. Moreover, overall, frozen cultures from log phase retained much higher viability compared to those from stationary phase (e.g., at cycle 6, the viability of WT from log and stationary cultures decreased by 1 log and 5 log, respectively). In frozen cultures from stationary phase, the survival of both mutants was slightly (not significantly) lower compared to WT. Whereas, in frozen log phase cultures, the *hsp1* mutant exhibited a significantly higher loss of viability compared to WT.

These data indicate that *L. plantarum* cells from stationary phase cultures are much more sensitive to freezing stress than those from log phase. Moreover, these findings entail a role for HSP1 in cryoprotection. The different effect of *hsp1* KO between log and stationary phase may reflect a different level of expression and/or accumulation of the corresponding protein and/or other cryotolerance-related factors.

### Knock Out of *hsp1* and *hsp3* Affects Biofilm Formation and Cell Surface Properties

**FIGURE 5:**
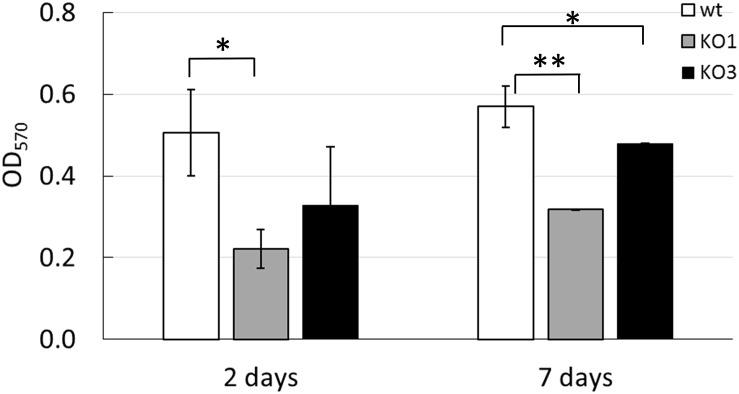
Biofilm formation by *L. plantarum* WCFS1 WT and its derivatives. Cultures were grown in MRS broth in 24-well cell culture plates at 30°C. Optical density at 570 nm (OD_570_) of the crystal violet-stained 2 days- and 7 days-biofilms is reported. Mean and standard deviations from at least three independent experiments, run in triplicates. Open bars, WT; gray bars, *hsp1* mutant (KO1); solid bars, *hsp3* mutant (KO3). ^∗∗^*P* ≤ 0.01; ^∗^*P* ≤ 0.05(Student *t*-test).

**FIGURE 6:**
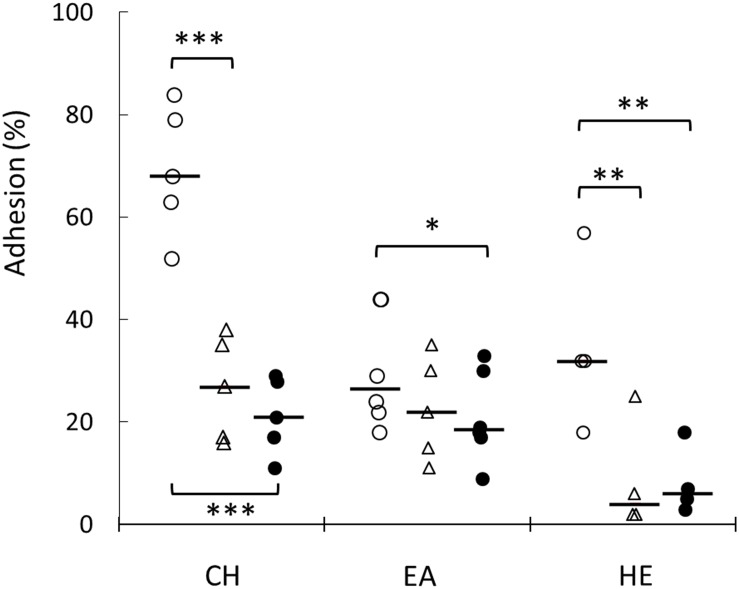
Evaluation of cell surface physicochemical properties of *L. plantarum* WCFS1 WT and its derivative mutants. Percentage of adhesion to chloroform (CH), ethyl acetate (EA), hexadecane (HE) of *L. plantarum* WT (open circles), *hsp1* mutant (open triangles) and *hsp3* mutant (solid circles). Individual mean data points from at least five experiments, run in triplicate, and the medians (bars) are shown. ^∗∗∗^*P* ≤ 0.001; ^∗∗^*P* ≤ 0.05; ^∗^*P* ≤ 0.1(Student *t*-test).

The formation of biofilm was investigated on a plastic surface (i.e., polystyrene). Both *hsp1* and *hsp3* mutants exhibited a decreased ability to form biofilm (Figure 5 and Supplementary Figure S1), indicating that absence of either sHSP affected directly or undirectly the process of biofilm development and suggesting an influence on some cell surface features associated to this phenotype. Relevant changes in physicochemical cell surface properties were confirmed by MATS analysis (Figure 6). The physico-chemical characters of the cell envelope were evaluated by determining the adhesion of *L. plantarum* WT, KO1, and KO3 mutants to three solvents with different chemical features, i.e., CH (monopolar and acidic), EA (monopolar and basic) and HE (apolar). Compared to WT, the mutants exhibited significantly lower affinity toward CH and HE, thus indicating that sHSP inactivation affects the electrostatic character (i.e., decreasing the electron-donating properties) and reduces the hydrophobicity of cell surface.

Taken together, these findings underpin the impact of sHSP on the adhesive and physicochemical surface properties of *L. plantarum* cells.

### HSP1 Could Be Involved in Membrane Stabilization

**FIGURE 7:**
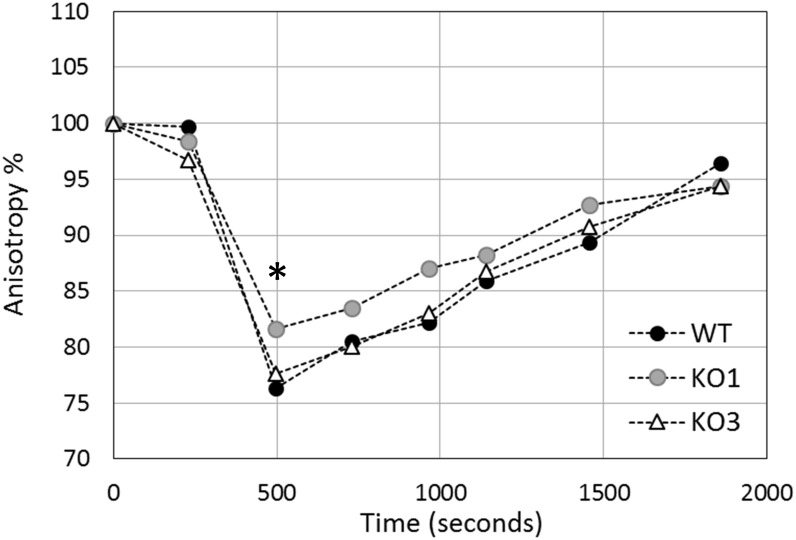
Evolution of *in vivo* membrane fluidity (fluorescence anisotropy percentage) in *L. plantarum* cells upon heat shock. Cells were heated at 44°C for 30 min and fluorescence anisotropy of inserted DPH was monitored continuously in *L. plantarum* (WT, solid circles), *hsp1* (KO1, gray circles) and *hsp3* mutants (KO3, open triangles). The graph reports averages of the values recorded over one minute, at characteristic time points. Results are expressed as percent anisotropy of the initial values (i.e., before stress exposure). The asterisk (^∗^) indicates the maximal level of membrane fluidity (reached approximately 8 minutes after stress start), which is significantly different between KO1 and WT (*P* ≤ 0.0001) and between KO1 and KO3 (*P* ≤ 0.01), as assessed by Student *t*-test.

To investigate whether *hsp1* and *hsp3* are involved in membrane stabilization, the variation in *L. plantarum* membrane fluidity was monitored in response to heat shock (Figure 7). For all the investigated strains (i.e., WT, KO1, and KO3 mutants), a sharp increase of membrane fluidification (i.e., a decrease of anisotropy) was observed by the first minutes of heat exposure. After about 8 min, a minimal anisotropy value was reached, which corresponds to a maximal level of membrane fluidity. Such a value was significantly higher in the KO1 strain, thus indicating a lower membrane fluidity. After 30 min, cells recovered approximately the initial level of membrane fluidity (i.e., 96, 94, and 94% of the initial value for WT, KO1, and KO3, respectively).

### Transcriptional Analysis in the Mutant Genetic Backgrounds

**FIGURE 8:**
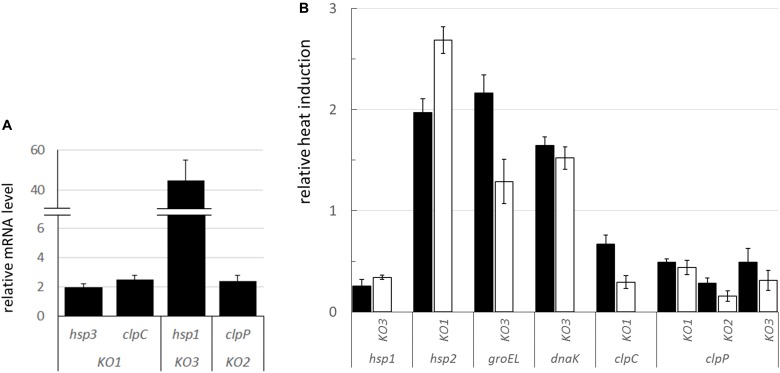
mRNA expression of stress-related genes in the *L. plantarum shsp* mutants compared to WT. **(A)** Relative mRNA expression of selected, significantly dysregulated stress genes under physiological condition (i.e., RNA extracted from log phase cultures grown at 30°C). mRNA levels were calculated relative to the transcript levels detected in the WT (i.e., WT level set at 1 for each gene). **(B)** Relative heat induction (i.e., RNA from log phase cultures grown at 30°C before and after heat challenge) of selected genes in the mutants compared to WT. mRNA levels, upon 30 min, solid bars, and 1 h, open bars, heat exposure (42°C), were calculated relative to the corresponding unstressed condition and normalized to the heat induction level observed in the WT (set at 1 for each gene and stress period, i.e., values above 1 indicate a higher induction relative to WT, values below 1 indicate a lower induction compared to WT). Data are means and SD from at least 2 independent experiments. Only genes whose transcription was significantly different compared to WT (*P* ≤ 0.05, Student *t*-test) are shown. *ldhD* and *tuf* were used as the internal control genes. KO1, *hsp1* mutant; KO2, *hsp2* mutant; KO3, *hsp3* mutant.

In order to evaluate whether the inactivation of *hsp1* and *hsp3* could induce a compensatory network to buffer against their mutations, the mRNA level of *shsp* and other stress-related genes (including those encoding chaperones and proteases, such as GroEL, DnaK, and Clp proteins) was analyzed in *L. plantarum* WT and *shsp* mutants (including the *hsp2* mutant) (Figure 8). The transcriptional analysis in bacterial cultures grown under optimal temperature conditions (30°C) revealed that some of the stress-related genes exhibited an altered expression relative to WT (Figure 8A). Particularly, compared to WT, *hsp1* was strongly up-regulated (*P* ≤ 0.05) in the KO3 mutant (but not in the *hsp2* mutant, data not shown). Moreover, *hsp3* and *clpC* were weakly upregulated in the KO1 mutant, while *clpP* mRNA level was more than 2-fold higher in the *hsp2* mutant compared to WT (Figure 8A).

By analyzing the transcriptional induction of stress genes upon heat shock exposure, we noticed that, among *shsp* genes, *hsp1* was much less activated in the KO3 mutant, compared to WT (Figure 8B). This is consistent with the already high mRNA level observed under physiological conditions (see above). Compared to the WT genetic background, a stronger heat-induced activation was observed for *hsp2* in the KO1 mutant, and to a minor extent, for *groEL* and *dnaK* in the KO3 mutant. In relation to *clp* genes, *clpC* induction was lower in the KO1 mutant, while *clpP* transcription was induced at a lower level in all the *shsp* mutants, including KO2, compared to WT.

## Discussion

In this study, a reverse genetics approach was undertaken to characterize the role of two small heat shock proteins in the stress response of *L. plantarum*, a versatile probiotic with remarkable relevance for both biomedical and industrial applications.

sHSP have been found in almost all organisms, from prokaryotes to eukaryotes. This ubiquity points to fundamental biological roles. Several organisms possess more than one sHSP. Yet, the number of sHSP-encoding genes is quite variable, being influenced by factors including lifestyle and genome size (Han et al., 2008; Guzzo, 2012). In eukaryotes, multiple sHSPs are common in many groups. Among microbes, those species living in more adverse and changeable environments tend to have larger repertoires of sHSPs. This hints to the crucial role of sHSPs in bacterial survival, facilitating the colonization of diverse and adverse niches. Lactobacilli typically have a single sHSP and *L. plantarum* is probably the only probiotic LAB that possesses such a large sHSP family (Han et al., 2008; Capozzi et al., 2011a). The presence of three different *shsp* genes might reflect its genome size (which is larger than the average for this group), as well as its ecological flexibility, thus allowing for an effective response to diverse environmental conditions.

In order to characterize the contribute of the single sHSPs to stress tolerance, we compared the growth of WT and KO strains under different types of abiotic stress conditions, focusing on those that characterize typical environmental niches of *L. plantarum*, including food-related matrices, biotechnological processing and the gut ecosystem. Accordingly, growth was monitored under heat, hyperosmotic and oxidative conditions, which may be encountered by microbial starters during food manufacture, as well as in presence of low pH and bile, which typically challenge food-delivered probiotics during transit along the gastro-intestinal tract (Mills et al., 2011; Fiocco et al., 2019). We observed that both mutants exhibited a similarly, (and significantly) impaired growth. In some cases (i.e., under acidic and oxidative conditions), growth was more affected in the *hsp1* mutant, suggesting a more relevant role for this particular sHSP. However, neither of the two sHSP seemed indispensable for coping with a particular type of stress, as growth capacity, even if inhibited to different extents, was retained for all the tested conditions. These data indicate that HSP1 and HSP3, unlike HSP2 (Capozzi et al., 2011b), are needed to improve growth under different stresses, particularly heat and bile, although they are not essential for survival. Besides, in relation to the range of stress considered, neither HSP1 nor HSP3 seemed specialized for counteracting a specific challenge. The significant growth impairment observed under control conditions (i.e., no stress), in both mutants, hints to a housekeeping role for *hsp1* and *hsp3*, which is consistent with their basal constitutive expression (Spano et al., 2004, 2005; Russo et al., 2012; Van Bokhorst-van de Veen et al., 2013) and with previous studies on other sHSP, including *L. plantarum* HSP2 (Capozzi et al., 2011b).

Growth under stress indicated that HSP1 and HSP3 are likely to fulfill largely overlapping functions. However, a diversified role for the two investigated sHSP emerged in relation to thermotolerance induction and to cryotolerance. Here we found that *L. plantarum*, like other lactobacilli (Teixeira et al., 1994; Desmond et al., 2002), can induce thermotolerance. Besides, *hsp3*, but not *hsp1*, is required to develop such thermal adaptation. Based on present and previous reports (Capozzi et al., 2011b), although heat shock survival was similar, of the three sHSP, only HSP3 was necessary to develop thermotolerance after pre-exposure to a mild heat challenge, and thus may be involved in the adaptation mechanism that protects microbial cells from subsequent intense stress (Papadimitriou et al., 2016).

As during pre-adaptation, the expression of stress-related functions is specifically stimulated (De Angelis et al., 2004), we can speculate that the different impact of the three *L. plantarum* sHSP on thermal adaptation might reflect their differential transcriptional regulation. Indeed, of the three genes, only *hsp3* was predicted to be transcriptionally controlled by the HrcA repressor alone (Spano et al., 2004; Fiocco et al., 2009, 2010). HrcA being a factor that inhibits the transcription of major chaperone genes, by binding to a conserved CIRCE (control of inverted repeat for chaperone expression) operator under non-stressed conditions (Van Bokhorst-van de Veen et al., 2013). Intriguingly, in the probiotic *Lactobacillus gasseri*, a HrcA-controlled *clp* gene (i.e., *clp L*) was also demonstrated to be essential to induce thermotolerance (Suokko et al., 2008).

Freezing is widely used to preserve starter and probiotic LAB for industrial applications, however it can severely damage microbial cells (Succi et al., 2007; Beal and Fonseca, 2015). Here we found that *L. plantarum* cells from logarithmic growth phase exhibited greater survival to freezing. The physiological conditions of cells are known to influence their stress tolerance. In *L. plantarum*, stationary phase cultures usually display higher stress tolerance compared with exponential phase ones (De Angelis et al., 2004; Parente et al., 2010). This reflects that some stationary phase-associated conditions, such as starvation and increasing acidity, activate general stress response genes, hence making cells more resistant to a range of adverse factors (De Angelis and Gobbetti, 2004), particularly to challenges such as heat, acid and oxidative stress (Parente et al., 2010; Papadimitriou et al., 2016). Among LAB, the cellular response to freezing, which is a combination of cold, ice, high osmotic pressure and dehydration stress, is still poorly characterized and might rely on additional and/or distinctive mechanisms and genes, possibly with a different pattern of activation. For instance, the transcript level of *L. plantarum cspC* and *cspP* (i.e., encoding cold shock proteins, CSP) was found to be high during early exponential growth at optimal temperature, while rapidly declining at the entry into stationary phase (Derzelle et al., 2000). An analogous *csp* transcriptional pattern was observed also in other microbial species (Ermolenko and Makhatadze, 2002). It is worth noting that CSPP overproduction was reported to enhance *L. plantarum* cryotolerance (Derzelle et al., 2003) and its transcriptional induction, upon cold adaptation, was associated with higher survival to freeze-thaw stress (Song et al., 2014).

Present data support a cryoprotective function for HSP1. Interestingly, the effect of *hsp1* inactivation on survival to freezing was associated to growth phase. A growth phase-dependent expression of the three *L. plantarum* sHSP was previously reported, indicating abundant *hsp1* (as well as *hsp3* and *hsp2*) mRNA levels during the exponential growth, along with a declining amount of the *hsp1* transcript in the stationary phase (Spano et al., 2005). This could account for the relevant and negligible consequence of *hsp1* KO that we observed on the cryotolerance of log and stationary phase cultures, respectively. Indeed, during the log phase, when *hsp1* is normally and abundantly expressed (in the WT), the lack of this protein (in the KO1 strain) might have major consequences on survival to freezing. By contrast, when, in the stationary phase, *hsp1* level is very low (or absent), the effect of its KO would become irrelevant for cryotolerance. The decreased survival of stationary phase cultures (compared to log phase ones) might also reflect the little amount of HSP1 available in this growth stage, thus corroborating a cryoprotective role for this sHSP. Notably, as we observed that *hsp3* is upregulated in the KO1 mutant, thus possibly fulfilling its roles (see below), we can infer that neither *hsp3* nor *hsp2*, being both expressed during the log phase, can compensate for the possible cryoprotection-specific activities of *hsp1*.

A link between cold stress and *L. plantarum* sHSP was supported by previous findings, including a strong induction of the *hsp1* transcript upon temperature downshift (Spano et al., 2004) and the cold-protective effect generated by *hsp1* overexpression (Fiocco et al., 2007). Indeed, interconnections between cold and heat adaptations mechanisms have been suggested in LAB (Papadimitriou et al., 2016). For instance, exposure to heat was demonstrated to cross-protect some LAB from cold and freezing (Broadbent and Lin, 1999; Walker et al., 1999). Furthermore, a few heat shock genes were found to be cold-inducible (Skinner and Trempy, 2001; Varcamonti et al., 2006). In phylogenetically distant organisms, including plants (Wang et al., 2017), insects (Rinehart et al., 2007) and yeast (Pacheco et al., 2009), sHSP have been shown to be involved in cold stress tolerance. Moreover, in bifidobacteria, the homologous over-expression of a sHSP resulted in increased resistance to low temperature (Khaskheli et al., 2015). However, as far as we know, a direct involvement of sHSP in cryoprotection has not been documented among LAB.

In *L. plantarum*, three cold-inducible genes, i.e., *cspL*, *cspP*, and *cspC*, encoding highly similar CSP, were identified previously (Derzelle et al., 2000, 2002). In adapting to cold stress, these different CSP seem to perform specific functions, which may relate to their ability to bind and stabilize RNA molecules (Derzelle et al., 2003; Rennella et al., 2017). HSP1 could possibly act as an antifreeze protein, especially if we consider the rigidifying effect of its inactivation on stressed cell membranes. However, additional experiments are necessary to unravel its molecular role and to determine whether HSP1 controls membrane fluidity by a direct interaction or by indirect mechanisms, e.g., by quality-controlling other proteins that can bind and stabilize the membrane. The recovery of membrane fluidity is much more linked to a physical reaction rather than to an enzymatic reaction or gene expression. However, an isomerization allowing the modification of fatty acids from *cis* to *trans* conformation may occur (Chu-Ky et al., 2005). This post-synthetic modification regulates the fluidity of bacterial membranes because the T_M_ of *trans* fatty acids is higher and they require less space in bacterial membranes (Denich et al., 2003).

Though growth was hindered under heat stress, the survival to an intense heat shock was similar between mutants and WT, and so was their capacity to prevent thermo-induced aggregation of cytoplasmic proteins. The level of aggregated proteins was generally low, both in WT and mutants, especially if we compare it with similar analyses in LAB species devoid of sHSP (Weidmann et al., 2017). This lack of remarkable consequences of *hsp1* and *hsp3* KO on both heat shock survival and protein thermoprotection prompted us to investigate whether a gene expression re-programming could occur in the mutants, thus possibly attenuating the consequences of the genetic loss. Transcriptional analysis of mutant and WT cultures revealed an altered expression of some stress genes, including *shsp*, both under basal conditions and in response to heat. Based on the observed pattern, we envisage that, under basal conditions, HSP1 may functionally compensate for HSP3 lack (and vice versa, even though to a much minor extent), possibly fulfilling highly similar (housekeeping) roles. By contrast, *hsp2* KO seems compensated by neither of the two sHSP mates. In this regard, it is worth highlighting that HSP1 and HSP3 share 45% amino acid sequence identity (61% similarity), while the level of identity relative to HSP2 is just 32% and 34% for HSP1 and HSP3, respectively (EMBOSS Needle alignment tool).

Both under normal conditions, and in response to heat, the transcriptional level of functions required for the active refolding and/or proteolytic elimination of denatured proteins was increased in some of the mutants compared to WT. sHSP normally interact and cooperate with such chaperone systems (Nakamoto and Vigh, 2007). Indeed, under stress conditions, energy-consuming chaperone machineries may be overloaded and the major role of sHSP is just to hold denatured substrates, in a soluble form, till the moment they can deliver them to downstream active chaperones. Our transcriptional data suggest that the lack of HSP3 and HSP1 could be compensated for by an increased production of some chaperone systems members (i.e., GroEL, DnaK), and augmented proteolytic activity and *hsp2* level, respectively. That would contribute to moderate the negative consequences of the loss of the sHSP holdase activity.

Knock out of *hsp1* was found to affect membrane fluidity in response to heat. In the KO1 strain, the maximal level of stress-induced fluidity was lower than in the WT, thereby indicating that the mutant holds a more rigid membrane, while HSP1 could (directly or indirectly) contribute to increase membrane fluidity. Intriguingly, such effect would be just opposite to that ascribed to other microbial sHSP with lipochaperone properties. Indeed, in most cases, the amphitropic microbial sHSP studied so far have been shown to increase the molecular order of the membrane, thus reducing its fluidity (Török et al., 2001; Coucheney et al., 2005; Maitre et al., 2012). Likewise, in *L. plantarum*, *hsp2* deletion resulted in increased fluidification upon ethanol stress, suggesting a membrane-stabilizing activity even for this sHSP (Capozzi et al., 2011b). Accordingly, it was speculated that lipochaperone sHSP could antagonize the heat-induced hyperfluidization of lipid bilayers (Nakamoto and Vigh, 2007). The putative membrane-fluidizing properties of *L. plantarum* HSP1 could pertain to its relevance in cryotolerance. In lactobacilli, a higher membrane fluidity has been associated to improved resistance to freezing (Gautier et al., 2013; Meneghel et al., 2017; Kwon et al., 2018). In fact, HSP1 could possibly contrast excessive membrane stiffening at low temperatures. In this regard, some antifreeze proteins from eukaryotic organisms have been proposed to protect and stabilize membranes by a direct physical interaction (Tomczak et al., 2002; Kar et al., 2016).

The involvement of HSP1 in membrane stabilization functions seems also corroborated by the impact of its KO on cell adhesive and surface physico-chemical properties. Most lactobacilli have basic and moderately hydrophobic cell surfaces (Muñoz-Provencio et al., 2009; Polak-Berecka et al., 2014). Cell surface characteristics are known to modulate LAB interactions with the environment, being relevant for adhesion and aggregation phenomena (Schär-Zammaretti and Ubbink, 2003) as well as for stress tolerance (Haddaji et al., 2015). MATS assay indicated that the KO of *hsp1* and *hsp3* resulted in a significant modification of the cell surface Lewis acid–base properties and hydrophobicity. In detail, relative to WT, both KO1 and KO3 mutant cells exhibited lower electron-donating properties and lower hydrophobicity. Analogous phenotypic changes were observed earlier in *L. plantarum hsp2* deletion mutant (Capozzi et al., 2011b). Surface hydrophobicity is thought to promote the initial phase of microbial adhesion (Schillinger et al., 2005). Accordingly, the lower hydrophobic character of the mutants correlated with a decreased biofilm-forming ability. Our findings indicate that sHSP could influence the chemical nature and/or the spatial organization of cell surface constituents, though further investigations are required to ascertain whether this reflects a specific (surface-located) activity or, rather, it is an indirect consequence.

Among LAB, only the small heat shock protein Lo18/HSP18, from *Oenococcus oeni*, has been extensively investigated, through heterologous and *in vitro* systems (Delmas et al., 2001; Coucheney et al., 2005; Maitre et al., 2012; Weidmann et al., 2017). Only recently, Darsonval et al. (2016) could confirm *in vivo*, using an antisense-RNA strategy, the role of this sHSP in thermotolerance and membrane protection. Our study is the first to demonstrate, *in vivo*, in a probiotic LAB, the relevance of two sHSP for improved growth under several stress conditions. Here we highlight the pleiotropic effects of *shsp* inactivation, including the induced compensatory network that may buffer against such genetic loss. Moreover, we suggest that HSP1 and HSP3 fulfill largely overlapping roles in stress tolerance, as well as in housekeeping functions, and observe their involvement in acquired thermotolerance, cryoprotection and membrane properties modulation. Further investigations, including complementation studies and analyses of the recombinant sHSP, shall be undertaken to shed light on such relationships and to demonstrate these functions more conclusively.

## Author Contributions

DF designed the experimental plan, performed most of the trials and wrote the manuscript. MA performed the experimental trials, analyzed the results and wrote the manuscript. AL, VC, and PR participated in the experiments and contributed to writing the manuscript. GS designed the experiments and read the final manuscript. AR, SW, and JG designed and performed part of the experiments, analyzed the results and read the final manuscript.

## Conflict of Interest Statement

The authors declare that the research was conducted in the absence of any commercial or financial relationships that could be construed as a potential conflict of interest.
