# Supplementary material for: The Phenotypic Analysis of Lactobacillus plantarum shsp Mutants Reveals a Potential Role for hsp1 in Cryotolerance
Source: Front Microbiol. 2019 Apr 24;10:838. doi: 10.3389/fmicb.2019.00838 (PMC6503756; doi:10.3389/fmicb.2019.00838)
Supplement: Supplementary file 1 [file Data_Sheet_1.PDF]

Supplementary Material

Table S1. Bacterial strains, plasmids and oligonucleotides used in this study

Name	Description[a] or sequence[b] (5'-3')	Reference, source or application
**Bacterial strains**		
E. coli DH10B	Cloning host	ThermoFisher Scientific
L. plantarum WCFS1	Single-colony isolate of *L. plantarum* NCIMB8826; originally isolated from human saliva	Kleerebezem et al., 2003
KO1	Cm^r; derivative of WCFS1 containing a lox66-P32-cat-lox71 cassette replacement of *hsp1* (*hsp1*::lox66-P32-cat-lox71)	This work
KO3	Cm^r; derivative of WCFS1 containing a lox66-P32-cat-lox71 cassette replacement of *hsp3* (*hsp1*::lox66-P32-cat-lox71)	This work
KO2	Cm^r; derivative of WCFS1 containing a lox66-P32-cat-lox71 cassette replacement of *hsp2* (*hsp1*::lox66-P32-cat-lox71)	Capozzi et al., 2011b
**Plasmids**		
pNZ5319	Cm^r Em^r;mutagenesis vector for gene replacements in *L. plantarum*	Lambert et al., 2007

pNZ5319-KOhsp1	Cm^r Em^r; pNZ5319 derivative containing homologous regions up- and downstream of lp_0129	This work
pNZ5319-KOhsp3	Cm^r Em^r; pNZ5319 derivative containing homologous regions up- and downstream of lp_3352	This work
**Oligonucleotides**		
Hsp1 B1ext-FOR	TCATCGAAAAAGGGAAGTTG	*hsp1* KO genomic control
Hsp1 B2ext-REV	GATTCATCAGGTCTTCGTGA	*hsp1* KO genomic control
Hsp3 B1extFOR	CTCCTGAACATCTTAACTGTAAAC	*hsp3* KO genomic control
Hsp3 B2extREV	GGCCAATGTCAAATACTCG	*hsp3* KO genomic control
FB1 hsp1	AA*GAGCTC*TGAATCGGAGAATGAGTCGG	*hsp1* KO vector construction
RB1 hsp1	AA*GAGCTC*AGCCATACTAACAATCCCCT	*hsp1* KO vector construction
FB2 hsp1	AA*ATTTAAAT*CCGAGCGCGAATGACGGTCA	*hsp1* KO vector construction
RB2 hsp1	AA*ATTTAAAT*GGCCCGCAGTTAACTCCGAC	*hsp1* KO vector construction
FB1 hsp3	AA*CTCGAG*GTTGTACTTCGCTGTCCAAG	*hsp3* KO vector construction

RB1 hsp3	AA*ATTTAAAT*GTCCCAATTCATCATATCGT	*hsp3* KO vector construction
FB2 hsp3	AA*ATTTAAAT*GCAGCTGCGGATACCCATCA	*hsp3* KO vector construction
FB2 hsp3	AA*ATTTAAAT*CTTCACGTCCACTGTTTCCG	*hsp3* KO vector construction
CAT For	TCAAATACAGCTTTTAGAACTGG	KO genomic control
CAT Rev	CCAGTAAATGAAGTCCATGGA	KO genomic control
Hsp1-rt F	AGGTTGATGTCCCTGGTATTG	qRT-PCR
Hsp1-rt-R	TAAAGACACCGTCAGCTTGG	qRT-PCR
Hsp2 rt F	CGGTGAAGTATGACGAA	qRT-PCR
Hsp2-rt-R	TTACCTTCGCTATCCCGCAAC	qRT-PCR
Hsp3-rt-F	CGCGAGTGAACGTCAAACTG	qRT-PCR
Hsp3-rt-R	ATCCGCAGCTGCCTTCTTT	qRT-PCR
ldhD rt-F	ACGCCCAAGCTGATGTTATATC	qRT-PCR
ldhD rt-F	AGTGTCCCACGAGCAAAGTT	qRT-PCR

Tuf rt- F	TTACTATCAACACTGCCCACG	qRT-PCR
Tuf rt-R	ACAACTAAGATCGCACCGTC	qRT-PCR
clpB rt-F	AGTTACCGGGCGTCCATACTG	qRT-PCR
clpB rt-R	GACTCAAAGCCGTCCTCAAG	qRT-PCR
clpC rt-F	ATC CTT TCC TCG CGA ATT TT	qRT-PCR
clpC rt-R	TGG CGT TCC TTC AGT CTT CT	qRT-PCR
clpE rt-F	TTTACCAACCCCAGCTTCAC	qRT-PCR
clpE rt-R	GGCAAAATCGATCCAGTGAT	qRT-PCR
clpP rt-F	TAG ATT GCT AAG CCG GCA GT	qRT-PCR
clpP rt-R	ATG TTA TCC GGT CCC ATT GA	qRT-PCR
groEL rt-F	ACCGGATTGAAGATGCTTTG	qRT-PCR
groEL rt-R	AACCAGCATTTTCAGCGATT	qRT-PCR

dnaK rt-F	TCAACCGTGTCACCCAAGTA	qRT-PCR
dnaK rt-R	TCCTTCAGTTGTGGCATTCA	qRT-PCR

[a] Cm^r, chloramphenicol resistant; Em^r, erythromycin resistant.

[b] Restriction sites are italicized
