# Supplementary material for: The Phenotypic Analysis of Lactobacillus plantarum shsp Mutants Reveals a Potential Role for hsp1 in Cryotolerance
Source: Front Microbiol. 2019 Apr 24;10:838. doi: 10.3389/fmicb.2019.00838 (PMC6503756; doi:10.3389/fmicb.2019.00838)
Supplement: Supplementary file 2 [file Data_Sheet_2.PDF]

frontiers

Supplementary Material

Table S2. Lag phase duration and maximal growth rates of *L. plantarum* wild-type (wt), *hsp1* (KO1) and *hsp3* (KO3) mutants, under control (i.e., unsupplemented MRS) and stress conditions. Related growth curves and conditions are as reported in figure 1.

Growth condition	Lag phase (h)			Maximum growth rates, μ_{max} (h^{-1}) [(a)]		
	WT	KO1	KO3	WT	KO1	KO3
unsupplemented MRS	3.5	3.5	3.5	0.390 ± 0.004	0.327 ± 0.018	0.323 ± 0.028
Bile 0.05 %	3.5	3.0	3.5	0.280 ± 0.020	0.200 ± 0.002	0.231 ± 0.024
Bile 0.2 %	7.5	13.5	14.0	0.335 ± 0.003	0.118 ± 0.001	0.131 ± 0.012
pH 4.0	3.0	3.5	3.5	0.282 ± 0.022	0.201 ± 0.023	0.186 ± 0.007
pH 4.5	3.0	3.5	4.0	0.311 ± 0.018	0.233 ± 0.038	0.267 ± 0.019
Diamide 0.5 mM	3.0	3.5	4.0	0.390 ± 0.027	0.331 ± 0.025	0.404 ± 0.053
Diamide 2 mM	3.5	5.0	5.0	0.342 ± 0.017	0.245 ± 0.013	0.248 ± 0.014
Diamide 4 mM	4.5	5.5	5.5	0.295 ± 0.005	0.176 ± 0.025	0.185 ± 0.005
EtOH 4%	3.5	4.5	4.0	0.304 ± 0.008	0.263 ± 0.025	0.296 ± 0.008
EtOH 6%	5.0	5.0	4.5	0.205 ± 0.022	0.118 ± 0.003	0.141 ± 0.019
NaCl 6%	6.0	5.0	6.5	0.265 ± 0.030	0.337 ± 0.002	0.257 ± 0.040
42 °C	3.0	3.5	3.5	0.276 ± 0.021	0.195 ± 0.009	0.196 ± 0.010

[(a)] mean value ± standard error
