# Supplementary material for: The Phenotypic Analysis of Lactobacillus plantarum shsp Mutants Reveals a Potential Role for hsp1 in Cryotolerance
Source: Front Microbiol. 2019 Apr 24;10:838. doi: 10.3389/fmicb.2019.00838 (PMC6503756; doi:10.3389/fmicb.2019.00838)
Supplement: Supplementary file 3 [file Data_Sheet_3.PDF]

Supplementary Material

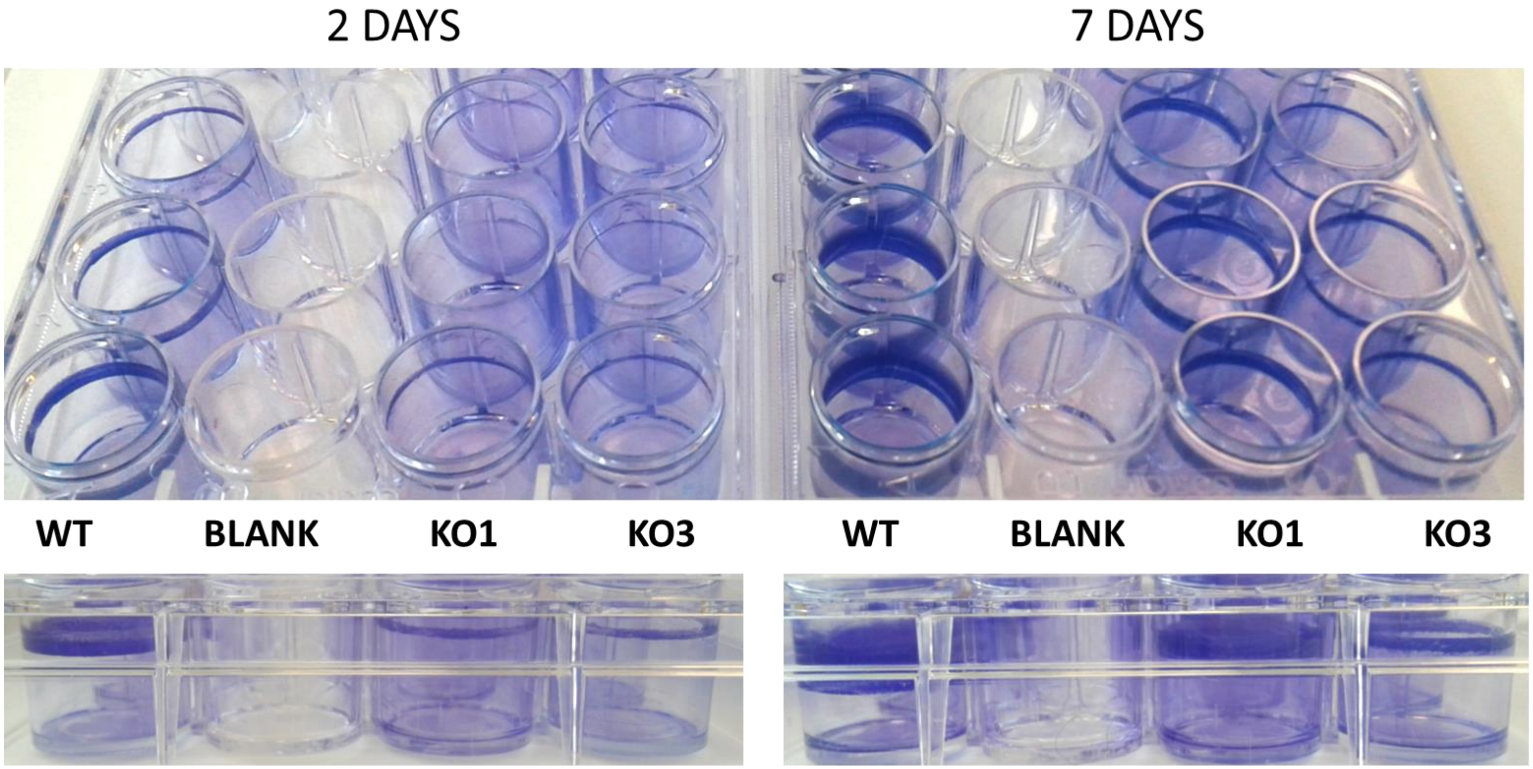

Figure S1. Representative picture of the crystal violet-stained biofilms formed by *L. plantarum* WCFS1 wild type (WT), *hsp1* mutant (KO1) and *hsp3* mutant (KO3), after 2 and 7 days culture, in MRS broth, in 24-well cell culture plates at 30°C.
